# Supplementary material for: Reconciling Mining with the Conservation of Cave Biodiversity: A Quantitative Baseline to Help Establish Conservation Priorities
Source: PLoS One. 2016 Dec 20;11(12):e0168348. doi: 10.1371/journal.pone.0168348 (PMC5173368; doi:10.1371/journal.pone.0168348)
Supplement: S1 Dataset — (ZIP) [file pone.0168348.s002.zip › Taxa/Serra Norte/SN_2013/N4E/Lista N4E-84.pdf]

CAVIDADE N4E-0084

Classe	Ordem	Fam/Outros	Gên/Outros	Espécie	S-U	U-S	Zon.
Arachnida	Acari	Mesostigmata		jovem	X		E
Arachnida	Acari	Veigaiidae		jovem	X		E
Arachnida	Amblypygi	Phrynidae	*Heterophrynus*	*longicornis*	2	2	E
Arachnida	Araneae	Caponiidae		jovem	X		E
Arachnida	Araneae	Oonopidae		jovem	X		E
Arachnida	Araneae	Pholcidae	*Leptopholcus*	sp.1		X	E
Arachnida	Araneae	Pholcidae	*Mesabolivar*	sp.1	X	X	E
Arachnida	Araneae	Pisauridae		jovem	1		E
Arachnida	Araneae	Salticidae		sp.2		X	E
Arachnida	Araneae	Theraphosidae		jovem		1	E
Arachnida	Araneae	Theridiidae		jovem		X	E
Arachnida	Araneae	Theridiosomatidae	*Plato*	sp.1	X	X	E
Arachnida	Opiliones	Sclerosomatidae	*Prionostema*	sp.1		1	E
Arachnida	Opiliones	Stygnidae	*Protimesius*	*gracilis*		1	E
Arachnida	Pseudoscorpiones	Chthoniidae		jovem	X		E
Diplopoda	Polydesmida	Fuhrmannodesmidae		jovem	X		E
Diplopoda	Polydesmida	Pyrgodesmidae		jovem	X		E
Entognatha	Collembola	Paronellidae		sp.1	X		E
Entognatha	Collembola	Poduromorpha		sp.1	X		E
Entognatha	Diplura	Japygidae		sp.1	X		E
Insecta	Blattodea	Blaberidae		jovem		1	E
Insecta	Coleoptera	Bostrichidae		sp.1	X		E
Insecta	Coleoptera	Staphylinidae		sp.46	X		E
Insecta	Diptera	Aulacigastridae		sp.		X	E
Insecta	Diptera	Chaoboridae		sp.	X		E
Insecta	Diptera	Chironomidae		sp.	X		E
Insecta	Diptera	Dolichopodidae		sp.	X		E
Insecta	Diptera	Drosophilidae		sp.	X		E
Insecta	Diptera	Phoridae	Metopininae	sp.	X		E
Insecta	Diptera	Sciaridae		sp.	X		E
Insecta	Diptera	Tipulidae	Tipulinae	sp.	X	X	E
Insecta	Heteroptera	Reduviidae	Reduviinae	sp.	1		E
Insecta	Heteroptera	Veliidae	*Microvelia*	sp.		X	E
Insecta	Homoptera	Cicadellidae		sp.1		X	E
Insecta	Homoptera	Cixiidae		sp.2		X	E
Insecta	Hymenoptera	Formicidae	*Camponotus*	sp.2		X	E
Insecta	Hymenoptera	Formicidae	*Eurhopalothrix*	sp.1	X		E
Insecta	Hymenoptera	Formicidae	*Pachycondyla*	sp.2	X	X	E
Insecta	Hymenoptera	Formicidae	*Paratrechina*	sp.1	X	X	E
Insecta	Hymenoptera	Formicidae	*Pheidole*	sp.2	X	X	E
Insecta	Hymenoptera	Formicidae	*Pheidole*	sp.3	X	X	E
Insecta	Hymenoptera	Formicidae	*Solenopsis*	sp.1	X		E
Insecta	Hymenoptera	Ichneumonidae		sp.1	X		E
Insecta	Isoptera	Termitidae	*Diversitermes*	sp.	X		E
Insecta	Isoptera	Termitidae	*Nasutitermes*	sp.	X	X	E
Insecta	Lepidoptera	Noctuoidea		sp.5	2	2	E
Insecta	Orthoptera	Phalangopsidae	*Paraclodes*	sp.	2	3	E
Insecta	Orthoptera	Phalangopsidae	*Phalangopsis*	sp.	5	10	E
Gastropoda	Pulmonata	Bulimulidae	*Naesiotus*	sp.	X		E

Malacostraca	Isopoda	Philosciidae		sp.1	X		E
Malacostraca	Isopoda	Platyarthridae		sp.1	X		E
Symphyla		Scutigerellidae		sp.	X		E
Turbellaria	Tricladida			sp.1		1	E
Amphibia	Anura	Strabomantidae	*Pristimantis*	cf. *fenestratus*	4		E
